# TCMBank-the largest TCM database provides deep learning-based Chinese-Western medicine exclusion prediction

**DOI:** 10.1038/s41392-023-01339-1

**Published:** 2023-03-31

**Authors:** Qiujie Lv, Guanxing Chen, Haohuai He, Ziduo Yang, Lu Zhao, Kang Zhang, Calvin Yu-Chian Chen

**Affiliations:** 1 Artificial Intelligence Medical Research Center, School of Intelligent Systems Engineering, Sun Yat-sen University, Guangzhou, China; 2 The Sixth Affiliated Hospital, Sun Yat-sen University, Guangzhou, China; 3 Center for Innovations and Biomedicine, Faculty of Medicine, Macao University of Science and Technology, Macao, China

**Keywords:** Predictive medicine, Drug safety

**Dear Editor**,

In the modernization of traditional Chinese medicine (TCM), two key aspects are determining the active ingredients in herbs and elucidating the mechanism of action between the active ingredients and targets. The construction of a comprehensive and highly-reliability TCM database is highly desirable.

Since its establishment in 2011, our TCM Database@Taiwan^1^ has been used extensively and heavily cited, and it also has been included in the ZINC database.^2^ Using natural language processing, we set up a knowledge graph and molecular signaling pathways to establish a TCM database, TCMBank (https://TCMBank.cn/), which extends from TCM Database@Taiwan and includes 9192 herbs, 61,966 ingredients, 15,179 targets, and 32,529 diseases. The updated TCMBank expanded the number of herbal ingredients from 32,364 to 61,966 (unduplicated), and two new data fields, targets, and diseases, have been added. The number of herbs with connection information is 9010, and the average number of connection edges of herbs is 16.05. The number of ingredients with connection information is 54,676, and the average number of connection edges of herbs is 5.26. TCMBank provides 3D structures of herbal ingredients in mol2 format and provides cross-reference links to external public databases, such as CAS, DrugBank, PubChem, MeSH, OMIM, DO, ETCM,^3^ HERB,^4^ etc. At present, TCMBank is the most comprehensive, downloadable, and largest non-commercial TCM database, and comparisons of data size between TCMBank and other TCM-related databases can be viewed in Fig. 1a. TCMBank provides a convenient website for users to freely explore the relationship between herbs, ingredients, gene targets, and related pathways or diseases (Fig. 1b). Figure 1c shows the process of establishing the TCMBank, including text mining strategy, intelligent document identification module, etc. All TCM-related information must be manually verified by volunteers at least twice to ensure the reliability of TCMBank data.

**Fig. 1:**
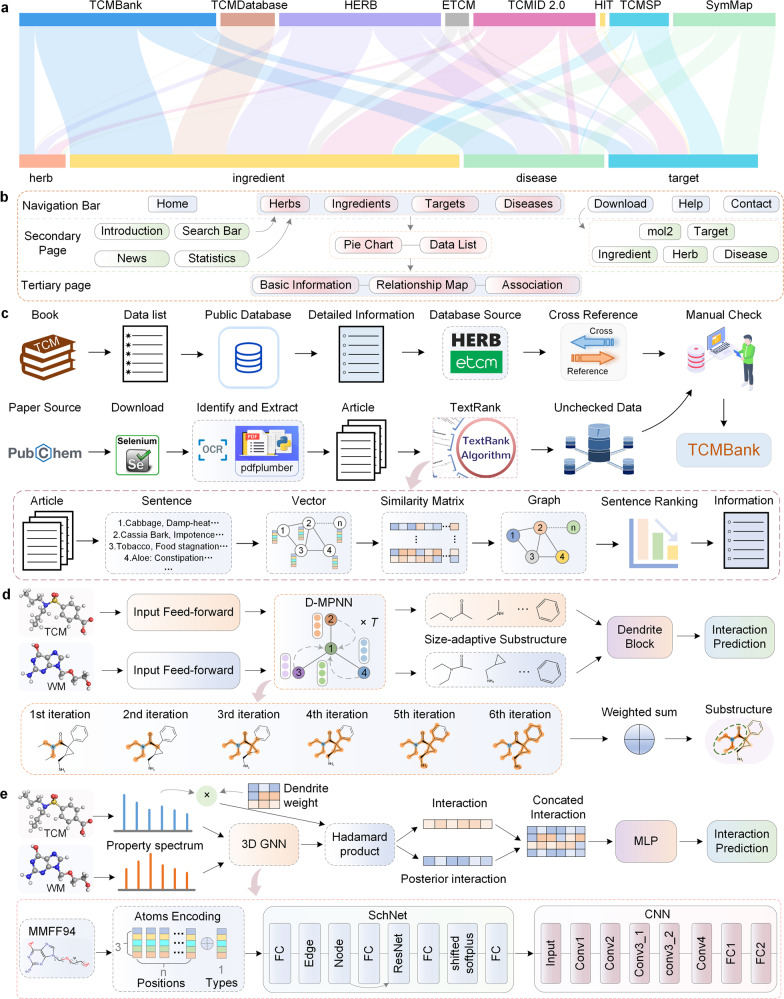
Comprehensive analysis of TCMBank, the largest database of traditional Chinese medicine. **a** The comparison of data sizes between TCMBank and other TCM-related databases, where TCMBank is the richest at herb, ingredient and disease. **b** The composition of TCMBank website, including the navigation bar, the home page, secondary page, and tertiary page. **c** A schematic diagram of the data processing framework and objectives in TCMBank. **d** Schematic diagram of adaptive substructure-aware module based on graph neural network for drug functional group extraction. **e** Mutual exclusion prediction of Chinese-Western medicines based on causal learning. TCM traditional Chinese medicine, WM western medicine, D-MPNN direct message passing neural network, 3D GNN three-dimensional graph neural network, MLP multilayer perceptron, MMFF94 Merck molecular force field, 1994 version, CNN convolutional neural network

Adverse reactions between Chinese-Western medicines can lead to increased medical costs and even death. It is estimated that more than 10% of patients need to take five drugs at the same time, and 20% of elderly patients need to take at least ten drugs at the same time, which greatly increases the medical risk caused by the mutual exclusion of Chinese-Western medicines. The identification of mutually exclusive reaction of Chinese-Western medicines mainly relies on biochemical assays in clinical. However, this process is very manpower and material consuming.

AI-based prediction of mutual exclusion of Chinese-Western medicines requires a large number of pairs of Chinese medicine and Western medicine with adverse reaction labels. There is a lack of mutual exclusion datasets for Chinese-Western medicines, while there are currently two real-world public drug–drug interactions (DDI) datasets: DrugBank and TWOSIDES. In previous works, we first proposed two models, 3DGT-DDI^5^ and SA-DDI,^6^ on the DDI datasets to predict the interaction between the two compounds. Supplementary Tables S1-S6 shows that 3DGT-DDI and SA-DDI achieve state-of-the-art performance on two public DDI datasets. Then, we extended the prediction results of the above two models to the prediction of mutual exclusion of Chinese-Western medicines. TCMBank provides the world’s largest herb-ingredient-target-disease mapping information. Benefiting from the big data drive of TCMBank, we used the DDI model to predict the mutual exclusion of Chinese-Western medicines for unsupervised learning. For a pair of traditional Chinese medicine and Western medicine, we query the active ingredients contained in TCM according to TCMBank. Assuming that all ingredients in the TCM do not have adverse reactions with Western medicine, it is determined that there is no mutually exclusive reaction between them. If one or more ingredients in the TCM have adverse reactions with Western medicine, they have a mutually exclusive reaction. In this way, we use an AI-assisted DDI prediction model to produce the prediction results of the mutual exclusion of Chinese-Western medicine.

The prediction results of the AI-assisted model have not been verified by actual clinical or biochemical tests. In the future, we will combine AI-assisted models for mutual exclusion prediction of Chinese-Western medicines, NLP and knowledge graph technology in text mining to develop a comprehensive database of combined Chinese-Western medicines. We will use IDIM module to search the mutual exclusion reaction of Chinese-Western medicine predicted by an AI-assisted model, and download, analyze the literature. Knowledge graph, keyword extraction and automatic summarization will be used to assist researchers to manually check the mutually exclusive information of Chinese-Western medicine contained in the literature. We will publish a comprehensive database of combined Chinese-Western medicines, which is a future work.

Another interesting future study will be to predict the mutually exclusive reaction of a group of multiple (more than two) Chinese-Western medicines. In the real world, the patient obviously intakes many more than two TCM or western medicine. This will require the development of new algorithms to consider the mutual exclusion of multiple drug combinations. Based on knowledge of medicinal chemistry, a drug is an entity composed of different functional groups/chemical substructures that determine their pharmacokinetic, pharmacodynamic properties, and the mutual exclusion of Chinese-Western medicine. We think that the interaction of substructure is regarded as the causal relationship of the interaction of Chinese-Western medicine, so as to establish a network of drug interactions or a network of interactions between multiple drugs (Fig. 1d), in which compounds as nodes and their causal relationships as edges. The nodes corresponding to all the ingredients in a TCM form a sub-network. We will predict whether TCM or Western medicine has mutual exclusion reaction according to whether there are edges between their corresponding sub-networks (Fig. 1e). Details of possible causal learning models are described in supplementary materials.

We developed TCMBank (https://TCMBank.cn/) to aggregate earlier studies dispersed in various forms of sources and create a comprehensive and reliable information system for Chinese medicine. TCMBank enables research on the molecular mechanism of herbal medicine and promotes the discovery of new drug molecules and corresponding potential molecular targets. The advantages of TCMBank include: (1) TCMBank is currently the largest downloadable and non-commercial database. (2) TCMBank provides up-to-date TCM-related information through continuous updates of the intelligent document recognition module. (3) TCMBank provides a large amount of herb/ingredient information with properties, physical and chemical properties, and 3D structure, as well as its target/disease information. We hope that TCMBank can meet the increasing needs for data resources related to TCM modernization and provide strong support for future advancement in the modernization of TCM.

## Supplementary information

Supplementary Materials

## Data Availability

TCMBank database is available at https://TCMBank.cn/. Any other information required to reanalyze the data reported in this paper are available upon request.
