# Supplementary material for: TCMBank-the largest TCM database provides deep learning-based Chinese-Western medicine exclusion prediction
Source: Signal Transduct Target Ther. 2023 Mar 31;8:127. doi: 10.1038/s41392-023-01339-1 (PMC10063611; doi:10.1038/s41392-023-01339-1)
Supplement: Supplementary file 1 — Supplementary Materials [file 41392_2023_1339_MOESM1_ESM.docx]

Supplementary Materials for

**TCMBank-the largest TCM database provides deep learning-based Chinese-Western medicine exclusion prediction**

**Qiujie Lv^1#^, Guanxing Chen^1#^, Haohuai He^1^****^#^, Ziduo Yang^1^, Lu Zhao^2^, Kang Zhang^3^ and Calvin Yu-Chian Chen^1∗^**

^1^ Artificial Intelligence Medical Center, School of Intelligent Systems Engineering, Sun Yat-sen University, Guangzhou 510275, China

^2^ The Sixth Affiliated Hospital, Sun Yat-sen University, Guangzhou 510275, China

^3^ Center for Innovations and Biomedicine, Faculty of Medicine, Macao University of Science and Technology, Macao, China

^#^ These authors contributed equally;

* e-mail: chenyuchian@mail.sysu.edu.cn

**This PDF file includes:**

Materials and Methods

Supplementary Tables S1 to S6

**Materials and Methods**

# The intelligent document identification module

Advancement in technology allows faster and purer isolation of new TCM ingredients. TCMBank has an intelligent document identification module (IDIM) to intelligently retrieve new information related to TCM or targets and continuously update the database. IDIM is divided into five stages: download, PDF parsing, intelligent retrieval, storage, and manual checking. First, TCMBank uses selenium web application testing tools to regularly download the latest documentation from PubChem. It runs directly in the browser, mimicking the operation of a real user, and does not burden the server. Then, the pdfplumber and pytesseract for optical character recognition (OCR) are used to parse the PDF and obtain detailed information about each text character, rectangle, and line. The optical structure recognition (OSRA), an open-source tool developed by the National Institutes of Health, automatically identifies and transforms the chemical structure in published reference figures into SMILES or structure data representation.

Next, IDIM extracts keywords and summaries through TextRank algorithm using only information from a single document (Figure 1d). We divide the published references into several constituent units (words, sentences) and perform preprocessing, such as word segmentation, part-of-speech tagging, and removal of stop words for each sentence. We then construct candidate keyword graphs and sentence similarity graphs for words and sentences, respectively, and use the voting mechanism to rank the important components in the text. The automatic summarization selects the top 10 keywords according to the sequence of importance voting, which selects for words and sentences with a higher importance in the text. PNG and SD are saved separately so that volunteers can check whether the structure and name information of the compound is correct in the database. Finally, we stored PDF, PNG, keywords, summary, SMILES, and other information in MySQL v5.7.36 and select the current storage time as the time-stamp. All data information will be manually validated at least twice before being incorporated into the TCMBank database.

# Illustrate the performance of intelligent document identification module with examples

Our IDIM uses AI techniques including selenium^1^, pdfplumber^2^, pytesseract^3^ for optical character recognition (OCR), optical structure recognition (OSRA)^4^, automatic summarization^5-7^, and keyword extraction for literature mining, and is divided into 5 stages: regular download, PDF parsing, intelligent retrieval, manual checking, and storage.

Here, we introduce the actual effect of each stage in IDIM. Selenium regularly downloads PDF literature from PubChem^8^. Only when the network is stuck, it may affect the efficiency of selenium download. The pdfplumber is used to parse PDF documents and get information about tree structure, text characters. Since the format and structure of the published literature are relatively fixed, it is easy to obtain the tree structure and text characters. The pytesseract is a python tool for optical character recognition (OCR) based on Tesseract-OCR engine from Google. The English recognition rate of pytesseract for pictures in scientific literature is more than 80%, which has met the daily needs of our IDIM. OSRA is used to identify chemical structures of molecular graph in the literature. The complete recognition accuracy of OSRA is about 70% - 80%, and the proportion of wrong one or two bonds is about 40%. The recognition accuracy of OSRA is slightly higher for multiple heterocycles, but the recognition accuracy is low for other slightly complex structures such as natural products and chiral molecules.

TextRank, an unsupervised algorithm for text processing based on graph ranking, is used to extract keywords and summaries from the text. For the automatic summarization task, we separately extract the key sentences from each section of the literature. Due to the existence of abstract section and conclusion section in the literature, we can often extract the key sentences of the summary or conclusion. The effect of abstract extraction of the entire literature is significant. In keyword extraction task, the keywords extracted by TextRank are combined with the existing keywords in the literature to form the keyword extraction results of the entire literature. The result of keyword extraction is relatively good, and the relationship between text can be more fully utilized. However, it may be greatly affected by word segmentation, text cleaning, and high-frequency words.

# Performance evaluation

The possible causal learning model is applied to the prediction of the mutual exclusion reaction of multiple Chinese-Western medicines, which is a future research work. At present, we have proposed two models, 3DGT-DDI^9^ and SA-DDI^10^, on the drug-drug interactions (DDI) datasets to predict the interaction between the two compounds.

3DGT-DDI is an attention-based intelligent framework composed of a 3D graph neural network and pre-trained text attention mechanism. We first performed 3D conformation of drug molecule SMILES through MMFF and then inputted it into the 3D graph network. We also used the feature extraction model of text description based on BERT^11^ to obtain the text information in DDI. Then, we extracted the position embedding vector of the drug name, to enhance the ability of context information feature extraction of the model. The position information of Drug1 and Drug2 was inputted into the embedding layer. Finally, we made feature concatenation and output the prediction of DDI.

We further presented a substructure-aware graph neural network, a message passing neural network equipped with a novel substructure attention mechanism and a substructure-substructure interaction module (SSIM) for DDI prediction (SA-DDI). The substructure attention was designed to capture size- and shape-adaptive substructures based on the chemical intuition that the sizes and shapes are often irregular for functional groups in molecules. The SSIM was used to model the substructure-substructure interactions by highlighting important substructures while de-emphasizing the minor ones for DDI prediction. Specifically, first, an input feed-forward module (i.e., a multi-layer perceptron) was utilized to nonlinearly transform the nodes for better feature representation. Then, the two molecular graphs are fed into a GNN (D-MPNN^12^ in our case) equipped with substructure attention to extract the size- and shape-adaptive substructures. Finally, the extracted substructures are fed into the SSIM to learn the substructure-substructure interactions from which the model makes a DDI prediction.

Details of two real-world public DDI datasets are as follows:

• DrugBank^13^ is a unique bioinformatics and cheminformatics resource that combines detailed drug data with comprehensive drug target information. It contains 1706 drugs with 191,808 DDI tuples. 86 interaction types describe how one drug affects the metabolism of another one. Each drug is represented as the simplified molecular-input line-entry system (SMILES). In the DrugBank dataset, each drug pair is only associated with a single type of interaction.

• TWOSIDES is constructed by Zitnik et al.^14^ after filtering and preprocessing the original TWOSIDES dataset^15^. It includes 645 drugs with 963 interaction types and 4,576,287 DDI tuples. As against the DrugBank dataset, these interactions are at the phenotypic level (i.e., headache, pain in the throat, and others) rather than metabolic.

The hyperparameters of 3DGT-DDI and SA-DDI are shown below:

Table S1. The selected values of hyperparameters for 3DGT-DDI.

| Hyperparameter | Selected value |
| --- | --- |
| Learning rate | 2e-6 |
| L2 weight decay | 1e-2 |
| Number of epochs | 200 |
| Mini-batch size | 6 |
| Hidden_size | 768 |
| Word embedding size | 64 |
| Max length | 128 |
| Number of Multiple hidden layers | 6 |
| Cutoff | 10 |
| Number of Layers | 6 |
| Number of Hidden | 128 |
| Number of Filters | 128 |
| Word position embedding size | 128 |

Table S2. The selected values of hyperparameters for SA-DDI.

| Hyperparameter | Selected value |
| --- | --- |
| Number of iterations | 10 |
| Number of hidden units for h_i_/h_ij_ | 64 |
| Learning rate | 1e-3 |

Tables S3 to S4 summarize the predictive performance of 3DGT-DDI, SA-DDI and previous models on the DrugBank and TWOSIDES datasets. The 3DGT-DDI, SA-DDI surpasses other baselines in the two datasets, which demonstrates the effectiveness of the proposed 3DGT-DDI and SA-DDI for DDI prediction.

Table S3 Comparison results (mean ± std in %) of the proposed models and baselines on the DrugBank dataset.

|  | **ACC** | **AUC** | **F1** | **Prec** | **Rec** | **AP** |
| --- | --- | --- | --- | --- | --- | --- |
| DeepCCI | 93.21 ± 0.27 | 97.03 ± 0.14 | 93.37 ± 0.27 | 91.26 ± 0.25 | 95.58 ± 0.47 | 95.95 ± 0.20 |
| MRGNN | 93.23 ± 0.19 | 97.31 ± 0.08 | 93.39 ± 0.17 | 91.14 ± 0.39 | 95.76 ± 0.09 | 96.45 ± 0.09 |
| SSI-DDI | 92.48 ± 0.21 | 97.01 ± 0.09 | 92.65 ± 0.20 | 90.59 ± 0.27 | 94.80 ± 0.19 | 96.11 ± 0.14 |
| GAT-DDI | 92.03 ± 0.18 | 96.28 ± 0.09 | 92.29 ± 0.16 | 89.47 ± 0.34 | 95.29 ± 0.21 | 94.64 ± 0.12 |
| GMPNN-CS | 95.31 ± 0.07 | 98.45 ± 0.01 | 95.40 ± 0.07 | 93.58 ± 0.14 | 97.29 ± 0.01 | 97.91 ± 0.02 |
| 3DGT-DDI | 91.50 ± 0.02 | 97.02 ± 0.11 | 96.18 ± 0.05 | 94.22 ± 0.03 | 97.35 ± 0.08 | 97.73 ± 0.18 |
| SA-DDI | **96.23 ± 0.10** | **98.80 ± 0.02** | **96.29 ± 0.09** | **95.02 ± 0.12** | **97.59 ± 0.07** | **98.36 ± 0.04** |

Table S4 Comparison results (mean ± std in %) of the proposed models and baselines on the TWOSIDES dataset.

|  | **ACC** | **AUC** | **F1** | **Prec** | **Rec** | **AP** |
| --- | --- | --- | --- | --- | --- | --- |
| DeepCCI | 75.16 ± 0.30 | 82.42 ± 0.31 | 77.03 ± 0.05 | 71.65 ± 0.68 | 83.31 ± 0.84 | 79.47 ± 0.35 |
| MRGNN | 85.39 ± 0.31 | 91.93 ± 0.20 | 86.46 ± 0.27 | 80.57 ± 0.37 | 93.28 ± 0.21 | 89.32 ± 0.22 |
| SSI-DDI | 82.21 ± 0.41 | 89.27 ± 0.38 | 83.11 ± 0.44 | 79.10 ± 0.31 | 87.56 ± 0.81 | 86.19 ± 0.41 |
| GAT-DDI | 67.32 ± 2.04 | 75.16 ± 2.47 | 63.70 ± 3.28 | 71.54 ± 2.31 | 57.62 ± 5.09 | 72.50 ± 2.45 |
| GMPNN-CS | 86.96 ± 0.03 | 92.94 ± 0.02 | 87.85 ± 0.04 | 82.20 ± 0.03 | 94.35 ± 0.10 | 90.38 ± 0.04 |
| 3DGT-DDI | 86.42 ± 0.21 | 92.82 ± 0.16 | 98.12 ± 0.08 | **82.71 ± 0.17** | 94.58 ± 0.13 | 89.12 ± 0.17 |
| SA-DDI | **87.45 ± 0.03** | **93.17 ± 0.04** | **88.35 ± 0.04** | **82.43 ± 0.02** | 95.18 ± 0.10 | **90.51 ± 0.08** |

# Ablation experiments

Table S5. Comparison results (mean ± std in %) of ablation study on the DrugBank dataset.

|  | **ACC** | **AUC** | **F1** | **Prec** | **Rec** | **AP** |
| --- | --- | --- | --- | --- | --- | --- |
| 3DGT-DDI-w/o-POS | 91.35 ± 0.11 | 96.58 ± 0.26 | 95.71 ± 0.23 | 93.14 ± 0.15 | 94.76 ± 0.15 | 96.68 ± 0.13 |
| 3DGT-DDI-w/o-3D | 90.68 ± 0.07 | 96.33 ± 0.07 | 94.59 ± 0.14 | 93.64 ± 0.17 | 96.35 ± 0.19 | 96.27 ± 0.11 |
| 3DGT-DDI | 91.50 ± 0.02 | 97.02 ± 0.11 | 96.18 ± 0.05 | 94.22 ± 0.03 | 97.35 ± 0.08 | 97.73 ± 0.18 |
| SA-DDI_MPNN | 94.27 ± 0.09 | 97.91 ± 0.03 | 94.37 ± 0.09 | 92.74 ± 0.14 | 96.06 ± 0.06 | 97.22 ± 0.04 |
| SA-DDI_noSA | 96.00 ± 0.07 | 98.72 ± 0.07 | 96.06 ± 0.07 | 94.63 ± 0.05 | 97.53 ± 0.09 | 98.25 ± 0.12 |
| SA-DDI_GMP | 93.54 ± 0.16 | 97.22 ± 0.06 | 93.62 ± 0.15 | 92.49 ± 0.43 | 94.79 ± 0.42 | 95.80 ± 0.07 |
| SA-DDI | **96.23 ± 0.10** | **98.80 ± 0.02** | **96.29 ± 0.09** | **95.02 ± 0.12** | **97.59 ± 0.07** | **98.36 ± 0.04** |

Table S6. Comparison results (mean ± std in %) of ablation study on the TWOSIDES dataset.

|  | **ACC** | **AUC** | **F1** | **Prec** | **Rec** | **AP** |
| --- | --- | --- | --- | --- | --- | --- |
| 3DGT-DDI-w/o-POS | 85.22 ± 0.17 | 91.71 ± 0.07 | 86.25 ± 0.09 | 80.34 ± 0.21 | 93.14 ± 0.21 | 87.72 ± 0.26 |
| 3DGT-DDI-w/o-3D | 85.93 ± 0.05 | 92.38 ± 0.14 | 86.73 ± 0.13 | 81.56 ± 0.13 | 92.36 ± 0.15 | 88.63 ± 0.15 |
| 3DGT-DDI | 86.42 ± 0.21 | 92.82 ± 0.16 | 87.32 ± 0.08 | **82.71 ± 0.17** | 94.58 ± 0.13 | 89.12 ± 0.17 |
| SA-DDI_MPNN | 87.23 ± 0.02 | 93.02 ± 0.03 | 88.17 ± 0.01 | 82.09 ± 0.05 | **95.23 ± 0.06** | 90.32 ± 0.03 |
| SA-DDI_noSA | 87.21 ± 0.09 | 93.03 ± 0.05 | 88.12 ± 0.10 | 82.23 ± 0.05 | 94.92 ± 0.17 | 90.33 ± 0.07 |
| SA-DDI_GMP | 75.32 ± 0.43 | 82.59 ± 0.66 | 78.14 ± 0.80 | 70.11 ± 0.70 | 88.35 ± 2.90 | 78.22 ± 0.74 |
| SA-DDI | **87.45 ± 0.03** | **93.17 ± 0.04** | **88.35 ± 0.04** | **82.43 ± 0.02** | 95.18 ± 0.10 | **90.51 ± 0.08** |

For the sake of exploring the role of each component in the DDI model, we observe the metric scores of different models by adding or removing other parts. Tables S5 to S6 summarize the predictive performance of ablation study of 3DGT-DDI, SA-DDI on the DrugBank and TWOSIDES datasets. “w/o” represents without this module. For 3DGT-DDI, we found that the effect is better when 3D graph is used than when position embedding is used, which may be owed to the 3D graph model carrying the 3D structure information of the drug, and the 3D-structure information is more abundant than the position information. After using position embedding and 3D graph models, the complete 3DGT-DDI model has a larger improvement, which is greater than the sum of one of position embedding and 3D graph. It indicated that the use of position embedding may play an auxiliary role in the prediction of the 3D graph and help the recognition of the 3D graph. Therefore, although the promotion of position embedding is smaller than 3D graph, it is still an important part of the model. Finally, the DDI model that uses all the components performed best. By comparing SA-DDI with SA-DDI_MPNN, SA-DDI_noSA, and SA-DDI_GMP, we found that the model can benefit from the proposed strategies for DDI prediction. The SA-DDI exceeds SA-DDI_GMP by a notable margin in two datasets, which reveals the validity of the proposed SSIM. Moreover, we found that the SA-DDI gains less improvement from the substruction attention. However, the substruction attention can reduce the over-smooth problem and improve the model's generalization ability.

# Future work

The release of TCMBank promotes the development of data-driven AI-assisted algorithm for identifying mutual exclusion of Chinese-Western medicines. A causal learning model is expected to solve the mutual exclusion prediction of multiple TCM or Western medicines. The GNN methods attempt to learn the correlation of each substructure with the mutual exclusion prediction of Chinese-Western medicines. According to the theory of causal learning, correlation does not equal causation. Building models using only correlations may have cognitive biases. The causal learning model regards the interaction of substructures as the causal relationship of mutual exclusion of Chinese-Western medicine, and determines the mutual exclusion of Chinese-Western medicine by analyzing the interaction of substructures. The substructure attention mechanism assigns different weights to substructures of different sizes, then the weighted sum of all substructures centered on an atom is the substructure of the adaptive size corresponding to that position.

D-MPNN can be used as the basic structure of the model. During the k-th iteration, D-MPNN extracts the substructure with radius k. For a bond feature $h_{ij}^{t}$ at $t$ step, we first obtain its graph-level representation $g^{t}\in\mathbb{R}^{h}$ by exploiting global topology pooling. Then, we assigned an attention score to each graph-level representation $g^{(t)}$ at the step $t$. To facilitate the comparison of coefficients between substructures of different sizes, softmax function is used to normalize $e^{(t)}$ all steps. Next, the edge representation $e_{i\to j}$ is given by the weighted sum of edge features in all steps, which captures substructure information with different radii. Finally, we return to node features by aggregating edge features.

After obtaining the adaptive substructure$h_{i}$, We use dendrite network to convert the substructure vectors into attribute spectra$h_{i}=W_{d}(h_{i}\circ h_{i})$, where $\circ$ is the Hadamard product. The attribute spectrum extends the representation of substructure to the response degree of different attributes. Assuming that ${p_{i}}^{(x)}$ and ${p_{j}}^{(y)}$ are the property spectra corresponding to the i-th substructure of Chinese medicine and the j-th substructure of Western medicine, respectively. And they are both represented by three attributes, namely ${p_{i}}^{(x)}=\left[ i_{1},i_{2},i_{3} \right]^{T}$ and ${p_{j}}^{(y)}=\left[ i_{1},i_{2},i_{3} \right]^{T}$, the interaction of the substructures $ssi_{ij}$ is expressed as $ssi_{ij}=W_{ssi}{p_{i}}^{(x)}\circ{p_{j}}^{(y)}$. The feature representation of the substructure interaction is the interaction of the substructure various properties, and this process simulates the interaction between the properties of the substructure. Furthermore, we design an independent factor loss ${loss}_{2}=-\frac{1}{N}\sum\sum_{i\neq j} {(cov(ss{im}_{i},ss{im}_{j}))}^{2}$ to decouple the relationship between substructural interactions, making it easier for the model to learn implicit causality.

**Supplementary Reference**

1. Selenium is a suite of tools for automating web browsers. https://www.selenium.dev/, accessed July 12 (2020).
2. pdfplumber: Plumb a PDF for detailed information about each text character, rectangle, and line. https://github.com/jsvine/pdfplumber, accessed October 18 (2020).
3. pytesseract: Python-tesseract is an optical character recognition tool for python. https://github.com/madmaze/pytesseract, accessed July 12 (2020).
4. Filippov, I.V., Nicklaus, M.C. Optical structure recognition software to recover chemical information: OSRA, an open source solution. *J Chem Inf Model.* **49**,3, 740–743 (2009).
5. Wen, Y., Yuan, H., Zhang, P. Research on keyword extraction based on word2vec weighted textrank. In: 2016 2nd IEEE International Conference on Computer and Communications (ICCC), 2109–2113 (2016). IEEE.
6. Mihalcea, R., Tarau, P. Textrank: Bringing order into text. In: Proceedings of the 2004 Conference on Empirical Methods in Natural Language Processing, 404–411 (2004).
7. Barrios, F., L ́opez, F., Argerich, L., Wachenchauzer, R. Variations of the similarity function of textrank for automated summarization. Preprint at https://arxiv.org/abs/1602.03606 (2016).
8. Kim, S. et al. Pubchem 2019 update: improved access to chemical data. *Nucleic Acids Res.* **47**, D1, 1102–1109 (2019).
9. He, H., Chen, G., Chen, C.Y.-C. 3DGT-DDI: 3D graph and text based neural network for drug–drug interaction prediction. *Brief. Bioinform.* **23**, 3, 134 (2022).
10. Yang, Z., Zhong, W., Lv, Q., Chen, C.Y.-C. Learning size-adaptive molecular substructures for explainable drug–drug interaction prediction by substructure-aware graph neural network. *Chem. Sci.* **13**, 29, 8693–8703 (2022).
11. Devlin, J., Chang, M.-W., Lee, K., Toutanova, K. Bert: Pre-training of deep bidirectional transformers for language understanding. Preprint at https://arxiv.org/abs/1810.04805 (2018).
12. Yang, K. et al. Analyzing learned molecular representations for property prediction. *J Chem Inf Model.* **59**, 8, 3370–3388 (2019).
13. Wishart, D.S. et al. DrugBank 5.0: a major update to the DrugBank database for 2018. *Nucleic Acids Res.* **46**, D1, 1074–1082 (2018).
14. Zitnik, M., Agrawal, M., Leskovec, J. Modeling polypharmacy side effects with graph convolutional networks. *Bioinformatics*. **34**, 13, 457–466 (2018).
15. Tatonetti, N.P., Ye, P.P., Daneshjou, R., Altman, R.B. Data-driven prediction of drug effects and interactions. *Sci Transl Med.* **4**, 125, 125–3112531 (2012).
